# A Velocity-Based Impedance Control System for a Low Impact Docking Mechanism (LIDM)

**DOI:** 10.3390/s141222998

**Published:** 2014-12-03

**Authors:** Chuanzhi Chen, Hong Nie, Jinbao Chen, Xiaotao Wang

**Affiliations:** 1 State Key Laboratory of Mechanics and Control Mechanical Structures, Nanjing University of Aeronautics and Astronautics, Nanjing 210016, China; E-Mails: hnie@nuaa.edu.cn (H.N.); chenjbao@nuaa.edu.cn (J.C.); 2 College of Astronautics, Nanjing University of Aeronautics and Astronautics, Nanjing 210016, China; E-Mail: wangxtao1977@nuaa.edu.cn

**Keywords:** low impact docking mechanism (LIDM), impedance control, velocity, capturing simulation

## Abstract

In this paper, an impedance control algorithm based on velocity for capturing two low impact docking mechanisms (LIDMs) is presented. The main idea of this algorithm is to track desired forces when the position errors of two LIDMs are random by designing the relationship between the velocity and contact forces measured by a load sensing ring to achieve low impact docking. In this paper, the governing equation of an impedance controller between the deviation of forces and velocity is derived, and simulations are designed to verify how impedance parameters affect the control characteristics. The performance of the presented control algorithm is validated by using the MATLAB and ADAMS software for capturing simulations. The results of capturing simulations demonstrate that the impedance control algorithm can respond fast and has excellent robustness when the environmental errors are random, and the contact forces and torques satisfy the low impact requirements.

## 1. Introduction

In order to dock two vehicles using a conventional mechanical docking assembly, the vehicles must be pressed together with sufficient forces to re-align the misalignment of the soft capture ring [1]. The action of forcing two vehicles together, particularly in space, might result in damages to one or both of the vehicles or sensitive systems [2]. Thus, a type of docking mechanism which provides low impact mating (*i.e.*, a low impact docking mechanism, abbreviated as LIDM) has been developed by NASA [3], ESA [4] and Chinese research institutions [5], respectively, in order to solve the problems noted above. In particularly, the LIDM has a reconfigurable control system [1], which permits a load sensing ring with an electromagnetic capture mechanism to perform a “soft” capture and mate two vehicles together. As a result, a specified desired force, the ideal contact force between two docking mechanisms, could be tracked during the capture process. Force tracking is the key to the success of capturing, which can be solved by compliance control. Impedance force tracking control is very practical in the field of robotic compliance control and the main concept is based on the impedance equation, which is the relationship between force and position/velocity error [6].

The impedance control technique proposed by Hogan [7] is one of the fundamental approaches for force tracking control of robot manipulators with constrained motion. Then, the performance of impedance control was improved and the application was expanded to other fields by many researchers [8–10]. Differing from the hybrid position and force control approach [11], impedance control regulates the force between a manipulator and the environment by defining the target impedance between position and contact force. The desired force is indirectly controlled by prespecifying a robot-desired displacement, which is determined by the stiffness and location of the environment [12]. One of the major practical difficulties with impedance control is that the environmental stiffness cannot be known precisely. Therefore accurate desired displacement cannot be designed to achieve accurate force control. In the past, many attempts have been made to solve this problem. Lasky and Hsia [13] employed a separate desired displacement modification control loop by using integral control. Lee [14] formulated the generalized impedance relationship between a motion error and a contact force error, and Seraji [15] generated a reference position using adaptive control. A neural network or fuzzy approach of force control was introduced to solve a number of uncertainty problems [16]. Besides, the accurate environment positions cannot be available in advance in impedance control, another difficulty for its implementation, and poorly estimated environmental information may cause poor force tracking results [17]. Especially for application of force control with random errors by applying a desired force, the exact estimation of environment position is difficult. Therefore, it is difficult to design an expected docking trajectory of two LIDMs, as the relative position errors of two vehicles are random.

In this paper, an impedance control algorithm based on velocity is proposed. The practical difficulties mentioned above of impedance control and the environmental stiffness that cannot be known precisely, are satisfactorily solved in this paper by introducing feedback information of contact forces and torques measured by force sensors. In addition, a desired velocity is introduced instead of the desired displacement since the precise desired displacement cannot be deduced from the position random errors between two LIDMs. A relational function between contact force and velocity of load sensing ring is designed in this algorithm to track desired force as well as the shape of contact surface. Besides, a filter is set in this algorithm as the control law of contact forces and torques of two LIDMs. The filter, which can directly affect each of the contact forces and torques, can affect the trajectory whereby the LIDM responds to the same external forces and torques by changing the filtering functions, and a group of suitable filtering functions may result in a better trajectory for the capturing process. In order to validate the algorithm, a series of simulations with MATLAB and ADAMS are presented. First of all, the governing equation between deviation of forces and velocity of an impedance controller is derived, and simulations are carried out to validate how impedance parameters affect the control characteristics. Then, the model of LIDM is built in ADAMS referring to the Low Impact Docking System of NASA, and a control module of LIDM is generated in ADAMS. Finally, the control system is built in MATLAB via the usage of the control module. The results of capturing simulations demonstrate that impedance control based on velocity is suitable for the LIDM, as well as, that the present algorithm is robust and the filter is necessary for the impedance control system.

## 2. Features of the LIDM and Dynamic Model

**Figure 1.**
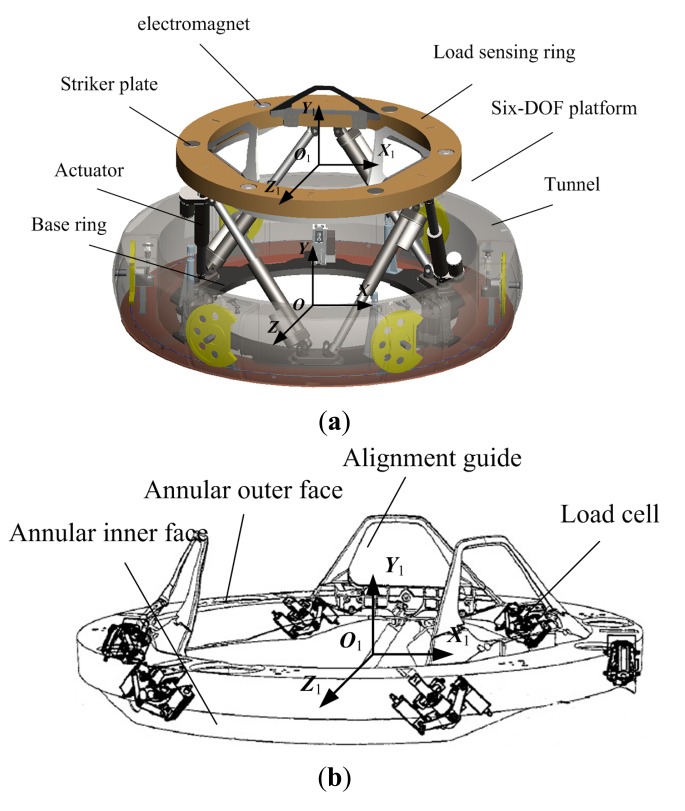
(**a**) The load impact docking mechanism (LIDM); (**b**) The LIDM load sensing ring.

The LIDM comprises a six-DOF platform, a tunnel and a control subsystem [1,2]. As shown in Figure 1a, the six-DOF platform is composed of a load sensing ring, a base ring, one or more electromagnets, one or more striker plates, a plurality of actuators, and a plurality of alignment guides. The load sensing ring and the base ring are coupled together using several actuators, base connection points and upper connection points. Structurally, the load sensing ring is comprised of an annular outer face, an inner face and a variety of load cells as shown in Figure 1b.

In addition, the six-DOF platform incorporates an active load sensing system so as to automatically and dynamically adjust the load ring during capture, instead of requiring significant force to push and realign the load ring. Unlike the mechanical trip latches that require a tripping force for capture, the LIDM uses electromagnets to achieve “soft” capture. Furthermore, the LIDM could also be controlled as a damper in lieu of interconnected linear actuators and separate load attenuation system, to eliminate the residual motion and dissipate the forces resulted from ramming two vehicles together. Therefore, the contact force and torque fluctuations can be maintained within a small range, and according to reference [18], it can be known that the maximum of contact forces and torques are not more than 450 N and 450 N·m, respectively. The dynamic model of the LIDM can be expressed as follows:

(1) $$\text{M}\left(\text{q}\right)\ddot{\text{q}}+\text{C}\left(\text{q},\dot{\text{q}}\right)\dot{\text{q}}+\text{g}\left(\text{q}\right)=\text{F}+F_{\text{f}}+F_{\text{e}}$$

where ***M***(***q***), ***C***(***q***), ***g***(***q***) represent the inertia matrix, centrifugal term and gravity term respectively; ***q*** represents the six-DOF generalized coordinate vector of the load sensing ring; ***F***, ***F***_f_ and ***F***_e_ represent the generalized driving force vector, generalized friction force vector and generalized external force vector respectively. In this paper, each generalized force is named “Force” below, which represents a six-dimensional vector and contains three forces and three torques along three axes of the coordinate. Similarly, the generalized displacement and the generalized velocity are named “Displacement” and “Velocity” respectively below. ***F*** is given from reference [19]:

(2) $$\text{F}=J^{\text{T}}\text{f}$$

where ***J*** represents the Jacobian Matrix, ***f*** = [*f*_1_ *f*_2_ *f*_3_ *f*_4_ *f*_5_ *f*_6_]^T^ represents the driving force matrix of actuators. In addition, there should be a certain relationship between external force and load cells as described previously, which can be expressed as follows.

Firstly, two coordinate frames are defined, namely ***O-XYZ*** and ***O***_1_-***X***_1_***Y***_1_***Z***_1_, which are fixed to the base ring and the load sensing ring respectively as shown in Figure 1. Then, the coordinates of load cell connection points ***a***_i_ and ***A***_i_ can be described in ***O***_1_-***X***_1_***Y***_1_***Z***_1_. Through the screw theory [20], the Jacobian Matrix ***J***_s_^T^ and the external Force in ***O***_1_-***X***_1_***Y***_1_***Z***_1_ can be obtained, as expressed below:

(3) $${\text{J}}_{s}^{T}=\left[\begin{array}{cccc} \frac{{\text{a}}_{1}-{\text{A}}_{1}}{\left|{\text{a}}_{1}-{\text{A}}_{1}\right|} & \frac{{\text{a}}_{2}-{\text{A}}_{2}}{\left|{\text{a}}_{2}-{\text{A}}_{2}\right|} & \cdots & \frac{{\text{a}}_{6}-{\text{A}}_{6}}{\left|{\text{a}}_{6}-{\text{A}}_{6}\right|}\\ \frac{{\text{A}}_{1}\times {\text{a}}_{1}}{\left|{\text{a}}_{1}-{\text{A}}_{1}\right|} & \frac{{\text{A}}_{2}\times {\text{a}}_{2}}{\left|{\text{a}}_{2}-{\text{A}}_{2}\right|} & \cdots & \frac{{\text{A}}_{6}\times {\text{a}}_{6}}{\left|{\text{a}}_{6}-{\text{A}}_{6}\right|} \end{array}\right]$$

(4) $$F_{\text{e}}^{\prime }=J_{s}^{\text{T}}f_{\text{s}}$$

where ***a***_i_ and ***A***_i_ represent the coordinates of the upper and the base connection points of load cells separately in ***O***_1_-***X***_1_***Y***_1_***Z***_1_, ***F***_e_ ′ represents the external Force vector in ***O***_1_-***X***_1_***Y***_1_***Z***_1_, ***f***_s_ is a vector composed of the values of six load cells. Thus, the external Force vector in ***O-XYZ*** can be described as follows:

(5) $$F_{\text{e}}=\text{R}F_{\text{e}}^{\prime }=\text{R}J_{s}^{\text{T}}f_{\text{s}}$$

where **R** is the rotation matrix transformed from ***O***_1_-***X***_1_***Y***_1_***Z***_1_ to ***O-XYZ***, and can be expressed as:

$$\text{R}=\left[\begin{array}{ccc} 1 & 0 & 0\\ 0 & \cos(\theta ) & -\sin(\theta )\\ 0 & \sin(\theta ) & \cos(\theta ) \end{array}\right]\;\left[\begin{array}{ccc} \cos(\beta ) & 0 & \sin(\beta )\\ 0 & 1 & 0\\ -\sin(\beta ) & 0 & \cos(\beta ) \end{array}\right]\;\left[\begin{array}{ccc} \cos(\alpha ) & -\sin(\alpha ) & 0\\ \sin(\alpha ) & \cos(\alpha ) & 0\\ 0 & 0 & 1 \end{array}\right]$$

where *α, β, θ* represent the Euler angles about axes ***Z***, ***Y***, ***X*** respectively. According to Equations (2) and (5), the dynamic model of LIDM can be rewritten as:

(6) $$\text{M}(\text{q})\ddot{\text{q}}+\text{C}(\text{q},\dot{q})\dot{q}+\text{g}(q)=J^{\text{T}}\text{f}+F_{\text{f}}+\text{R}J_{s}^{\text{T}}f_{\text{s}}$$

## 3. The LIDM Control System

### 3.1. The Flexible Model of LIDM

**Figure 2.**
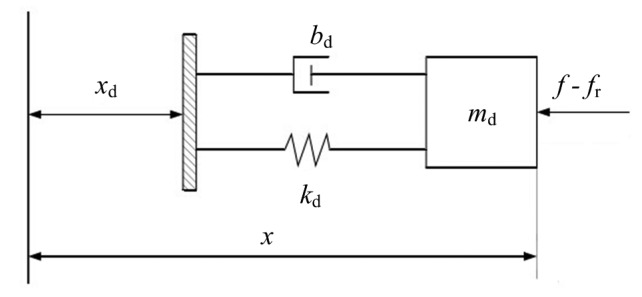
The flexible model of LIDM.

The LIDM is a rigid structure system, but when controlled by a force tracking control system, it can be treated as a flexible system with spring and damper characteristics. Therefore, the LIDM is supposed to be a mass-spring-damper system. The supposed model along one direction is shown in Figure 2, where *m*_d_, *k*_d_, and *b*_d_ represent the mass, stiffness and damping respectively, *x* and *x*_d_ represent the actual displacement and the desired displacement respectively, *f*_r_ and *f* represent the desired force and the external force respectively. The flexibility of supposed model is determined by parameters *m*_d_, *k*_d_, and *b*_d_, which are selected based on Equation (6). According to the flexible model of LIDM, the governing equation of an impedance controller can be built as descried in next section.

### 3.2. Impedance Controller Based on Velocity

The impedance control is based on the concept that it is neither position nor force, but the dynamic relationship between them that should be controlled [6]. In this section, ***q*** is replaced by ***X*** for the purpose of mathematical tractability, where ***X*** represents the six-DOF generalized coordinate vector of load sensing ring. According to Figure 2, the relation is an impedance equation given by:

(7) $$M_{\text{d}}(\ddot{\text{X}}-{\ddot{\text{X}}}_{\text{d}})+B_{\text{d}}(\dot{\text{X}}-{\dot{\text{X}}}_{\text{d}})+K_{\text{d}}(\text{X}-X_{\text{d}})=\text{E}$$

where ***E*** represents the deviation of Force between the external Force and the desired Force, while ***E*** = ***F***_e_ − ***F***_r_, in which ***F***_r_ represents the desired Force; ***X*** and ***X***_d_ represent the actual Displacement and the desired displacement, respectively; ***M***_d_, ***B***_d_ and ***K***_d_ are respectively 6 × 6 constant-positive-diagonal matrices of desired inertial, damping and stiffness. Apparently, a desired Displacement is necessary for an impedance control system based on the position from Equation (7). However, the desired Displacement of load sensing ring cannot be obtained, since the initial docking conditions of LIDM are random. Therefore, an impedance control method based on velocity is introduced to solve this problem. Thus, the relationship between force and velocity can be expressed as follows:

(8) $$M_{\text{d}}(\dot{\text{V}}-{\dot{\text{V}}}_{\text{d}})+B_{\text{d}}(\text{V}-V_{\text{d}})+K_{\text{d}}\int (\text{V}-V_{\text{d}})\text{d}t=\text{E}$$

where *V* and *V*_d_ represent actual Velocity and desired Velocity, respectively. When the initial docking conditions are zero, Equation (8) can be expressed in the Laplace domain as follows:

(9) $$M_{\text{d}}s\left[\text{V}(s)-{\text{V}}_{d}(s)\right]+B_{\text{d}}\left[\text{V}(s)-V_{\text{d}}(s)\right]+K_{\text{d}}\left[\text{V}(s)-V_{\text{d}}(s)\right]/s=\text{E}(s)$$

Let:

(10) $$V_{\text{f}}(s)=\text{V}(s)-V_{\text{d}}(s)$$

where *V*_f_ (*s*) represents the Velocity offset, thus, the *V*_f_ (*s*) can be obtained from Equation (9), and expressed as follows:

(11) $$V_{\text{f}}\left(\text{s}\right)=\frac{E\left(s\right)s}{M_{\text{d}}s^{2}+B_{\text{d}}s+{\text{K}}_{d}}$$

**Figure 3.**
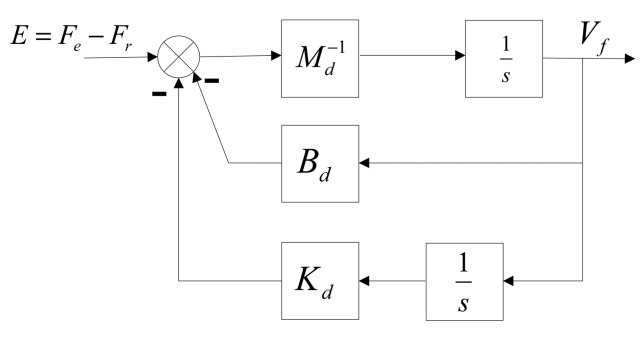
The structure diagram of the impedance control compensator.

According to Equation (11), the structure diagram of the impedance controller is established as shown in Figure 3. If there is no external Force on the LIDM (e.g., ***F***_e_ = 0) and the desired Force is assumed to equal to zero (e.g., ***F***_r_ = 0), the motion of load sensing ring follows the desired Velocity. Conversely, the motion of the load sensing ring is controlled by the correction of Velocity and the desired Velocity simultaneously.

According to Equation (11) and Figure 3, the dynamic relationship between deviation of Force and correction of Velocity can be adjusted to adapt to external environment by changing the impedance parameters. Detail information about how impedance parameters affect control characteristics will be shown in the next section.

### 3.3. The Influences of Impedance Parameters on Control Characteristics

**Figure 4.**
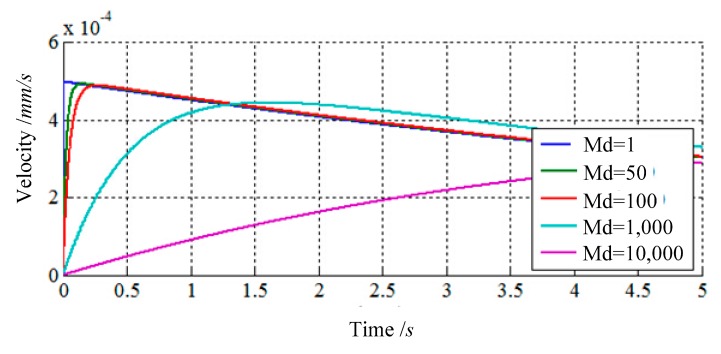
The responses of impedance control compensator with changing *M*_d_.

**Figure 5.**
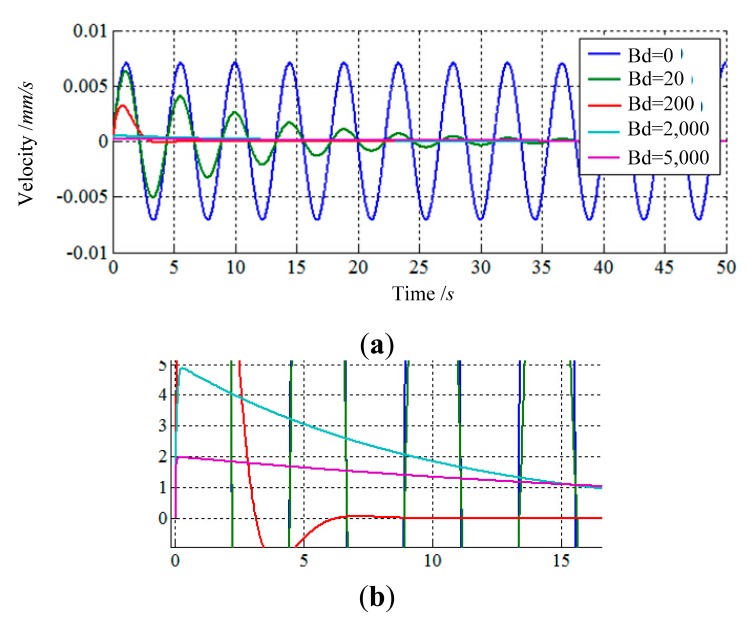
(**a**) The responses of impedance control compensator with changing *B*_d_; (**b**) The partial enlarged detail of Figure 5a.

**Figure 6.**
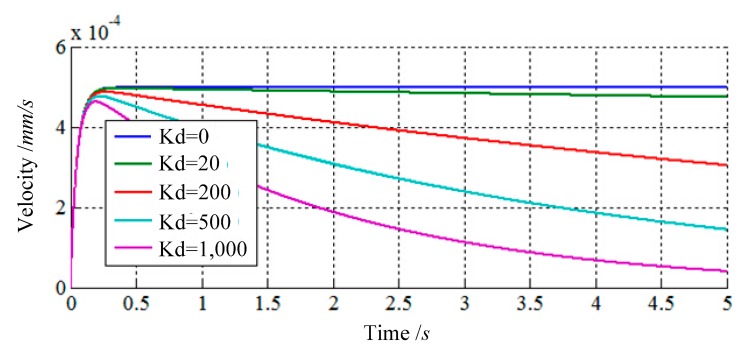
The responses of impedance control compensator with changing *K*_d_.

The purpose of impedance control based on velocity is to achieve an ideal dynamic relationship between the velocity of the load sensing ring and external forces by choosing a set of suitable impedance parameters. Thus, it is necessary to research how to choose the suitable impedance parameters. In this section, three simulations in one direction are presented to introduce how the impedance parameters affect control characteristics. The input function of simulations is a step function, and the results are shown in Figures 4, 5 and 6.

The first simulation shows how inertial parameter ***M***_d_ affects control characteristics by changing the value of ***M***_d_ and keeping ***K***_d_ and ***B***_d_ constant, while ***K***_d_ = 200 N/mm, ***B***_d_ = 2000 Kg/s. According to Figure 4, the inertial parameter ***M***_d_ of the impedance controller primarily affects the reaction rate of the responses. If a lower value is selected for ***M***_d_, there will be a rapid response to external forces, but it will result in a larger acceleration on actuators simultaneously. On the contrary, if a larger value is selected for ***M***_d_, the response rate of the impedance controller would be slow, resulting in a stronger external force.

The second simulation shows how damping parameter ***B***_d_ affects control characteristics by changing the value of ***B***_d_ and keeping ***K***_d_ and ***M***_d_ constant, while ***K***_d_ = 200 N/mm, ***M***_d_ = 100 Kg. According to Figure 5, the damping parameter of the impedance controller primarily affects the peak and regulation time of responses. With the increase of ***B***_d_, the peak of response decreases, and the regulation time reduces first and then increases. When the value of ***B***_d_ is equal to zero, the response of the impedance controller is undamped oscillation and the regulation time approaches to infinity. The vibration of response is not suitable for the compliance of LIDM, because it could result in an enormous external force.

The third simulation shows how the stiffness parameter ***K***_d_ affects the control characteristics by changing the value of ***K***_d_ and keeping ***M***_d_ and ***B***_d_ constant, while ***M***_d_ = 100 Kg, ***B***_d_ = 2000 Kg/s. According to Figure 6, it can be concluded that the stiffness parameter ***K***_d_ of the impedance controller primarily affects the attenuation of response. When ***K***_d_ is equal to zero, there is no attenuation. Additionally, the decay rate of response would increase, if the value of ***K***_d_ increases.

According to the results of simulations, if one or more of contact forces or/and torques are more than the maximum of requirements of low impact, a smaller ***M***_d_ should be selected to decrease them by increasing the reaction rate of the responses. The damping parameter ***B***_d_ must be maintained in a certain range where the oscillation of LIDM can be eliminated. When one or more of contact forces or/and torques are over the maximum of requirements of low impact, a greater ***B***_d_ should be selected to decrease them by increasing the peak of responses. The stiffness parameter ***K***_d_ of the impedance controller primarily affects the attenuation of responses. It is positive to maintain two LIDMs constant contact with each other during the process of capturing when the responses can decay (e.g., ***K***_d_ > 0) in this control system. However, some larger internal forces of LIDM may be caused, if one greater ***K***_d_ is selected.

### 3.4. The LIDM Model in ADAMS and the Control Module in MATLAB

**Figure 7.**
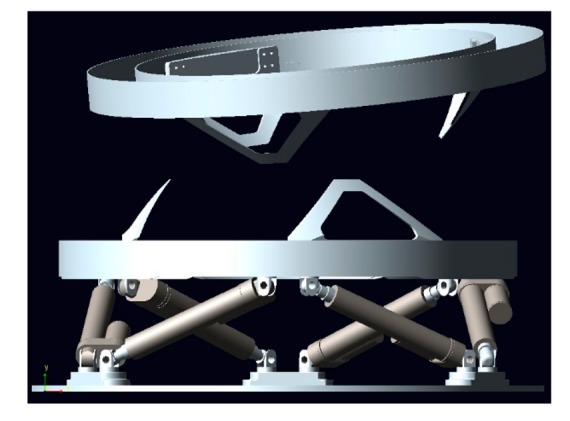
The LIDM model built in ADAMS.

The model of LIDM built in ADAMS is shown in Figure 7. In order to simplify the simulations, the inoperative parts of LIDM are removed. The ADAMS model comprises two docking assemblies, the active docking assembly (below in Figure 7) and the passive docking assembly (above in Figure 7).

The active docking assembly is composed of base ring, actuators and load sensing ring. The passive docking assembly is only comprised of annular outer face and alignment guides. Contact forces and torques of two docking assemblies, delivered to the impedance controller, can be measured by load sensing ring in real-time. The actuators can receive velocity signals from impedance controller to adjust the position and posture of load sensing ring. Besides, the initial docking conditions of two LIDM can be set through adjusting the position and posture of passive docking assembly.

**Figure 8.**
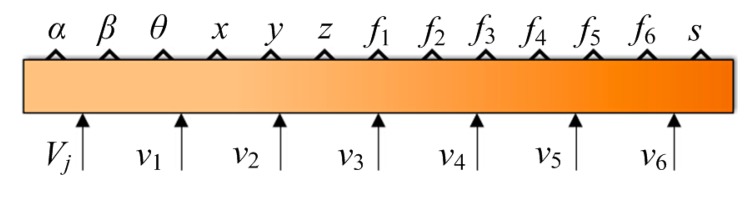
The control module of the LIDM in MATLAB.

The ADAMS model can be used to generate a control module through “controls” of soft ADAMS. The control module of LIDM in MATLAB is shown in Figure 8. *V*_j_, *v*_1_, *v*_2_, *v*_3_, *v*_4_, *v*_5_, *v*_6_ are input variables of the control module, where *V*_j_ represents the relative closing speed of two LIDMs, and *v*_1_, *v*_2_, *v*_3_, *v*_4_, *v*_5_, *v*_6_ represent the driving velocities of six actuators. *α, β, θ, x, y, z, f*_1_, *f*_2_, *f*_3_, *f*_4_, *f*_5_, *f*_6_ , *s* are the output variables, where *f*_1_, *f*_2_, *f*_3_, *f*_4_, *f*_5_, *f*_6_ represent the values of the load cells, and *s* represents the relative distance single of two LIDMs, through which completion of capture tasks can be detected.

### 3.5. The Components of the Control System

**Figure 9.**
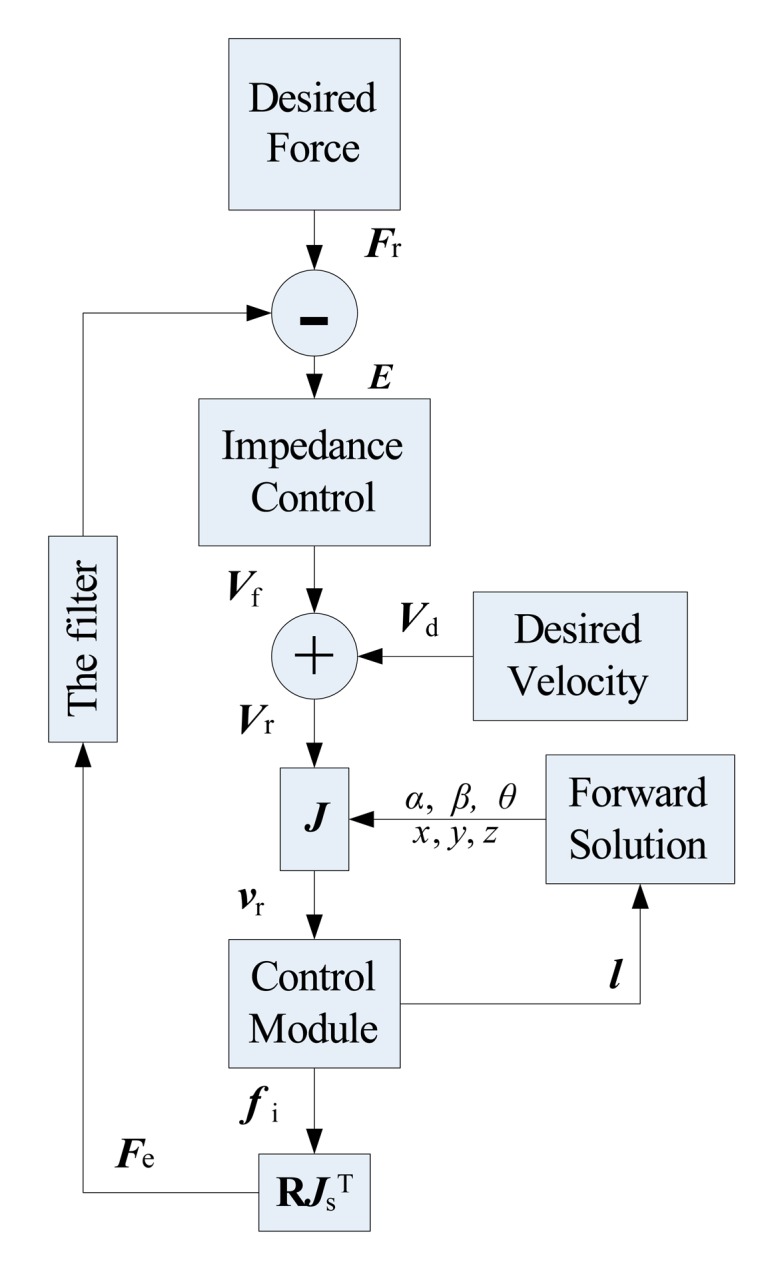
Components of the control system.

The control system built for LIDM consists of control modules, desired Force, impedance control, forward solution, Velocity transformation ***J***^T^, desired velocity, filter and other modules as shown in Figure 9.

The desired Force provides desired forces and torques along three Cartesian axes, which represents the ideal interaction Force between the two LIDMs. The input of impedance control part is the difference between actual external Force ***F***_e_ and desired contact Force ***F***_r_. The output is Velocity offset ***V***_f_. ***V***_r_ represents the actual Velocity of load sensing ring in Cartesian-space which has been adjusted to adapt to the docking environment, while ***V***_r_ = ***V***_f_ + ***V***_d_. ***v***_r_ is driving velocity vector of six actuators, converted from velocity ***V***_r_ by equation ***v***_r_ = ***J V***_r_. The length vector of six actuators ***l*** can be converted to the position and attitude angles (*i.e., x, y, z, α, β, θ*) of load sensing ring in Cartesian coordinates by forward solution. In addition, the control module is generated in ADAMS, which is the interface between ADAMS and MATLAB, and contacts the LIDM model in ADAMS with control system in MATLAB. Therefore, the control module can be seen as a MATLAB module that has the same effect on the model in ADAMS. In order to reduce computational complexity, *x, y, z, α, β, θ* can be directly obtained from control module, thus the forward solution in this simulation is unnecessary. However, this method is not suitable for physical test. Besides, the values of load cells can be converted to the contact Forces ***F***_e_ in Cartesian-space through Jacobian matrix **R*J***_s_^T^.

A filter is set in the feedback loop to control the values of external Forces passing the filter. When any one of the absolute values of input forces and torques of filter (except *F*_ey_) is less than or equal to critical value, the output of it is equal to zero, on the contrary, the output value is equal to input. In addition, whatever the input value is, the output of filter about *F*_ey_ is always equal to zero. The filter can not only remove some interference from environment, but also control the sensitivity about forces and torques along three Cartesian axes, which determines who responses to the same input data first. Without filter, the docking of two LIDMs may not be successful.

## 4. Simulation and Analysis

### 4.1. Simulation Setups

**Figure 10.**
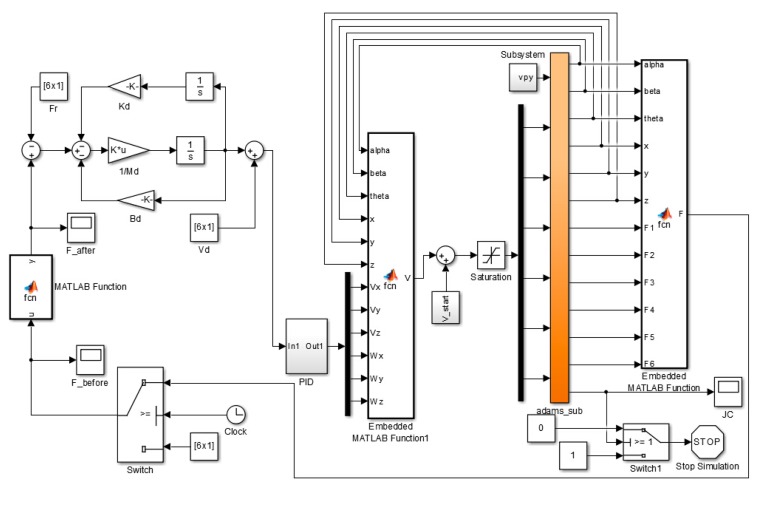
The control system built in MATLAB.

The control system is built in MATLAB using the control module, as shown in Figure 10. The sample time of simulation is 0.001 s. The simulation is mainly built to validate the performance of impedance control based on velocity in uncertain environment and the reasonableness of LIDM model. The impedance parameters are given by:

$$\begin{array}{c} \begin{array}{cc} {\text{M}}_{d}=\left[\begin{array}{cccccc} 100 & 0 & 0 & 0 & 0 & 0\\ 0 & 100 & 0 & 0 & 0 & 0\\ 0 & 0 & 100 & 0 & 0 & 0\\ 0 & 0 & 0 & 300 & 0 & 0\\ 0 & 0 & 0 & 0 & 300 & 0\\ 0 & 0 & 0 & 0 & 0 & 300 \end{array}\right], & {\text{B}}_{d}=\left[\begin{array}{cccccc} 2000 & 0 & 0 & 0 & 0 & 0\\ 0 & 2000 & 0 & 0 & 0 & 0\\ 0 & 0 & 2000 & 0 & 0 & 0\\ 0 & 0 & 0 & 2000 & 0 & 0\\ 0 & 0 & 0 & 0 & 2000 & 0\\ 0 & 0 & 0 & 0 & 0 & 2000 \end{array}\right] \end{array}\\ {\text{K}}_{d}=\left[\begin{array}{cccccc} 200 & 0 & 0 & 0 & 0 & 0\\ 0 & 200 & 0 & 0 & 0 & 0\\ 0 & 0 & 200 & 0 & 0 & 0\\ 0 & 0 & 0 & 200 & 0 & 0\\ 0 & 0 & 0 & 0 & 200 & 0\\ 0 & 0 & 0 & 0 & 0 & 200 \end{array}\right] \end{array}$$

The structure parameters of LIDM are given by Table 1, where ***a***_i_ and ***A***_i_ represent the coordinates of upper connection points and base connection points of load cells separately in ***O***_1_-***X***_1_***Y***_1_***Z***_1_, ***b***_i_ represents the upper connection points of actuators in ***O***_1_-***X***_1_***Y***_1_***Z***_1_, while ***B***_i_ represents the base connection points of actuators in ***O-XYZ***.

The filtering functions are expressed as Equations (12):

(12) $$F_{ei}=\{\begin{array}{cc} 0 & \left|F_{ei}\right|\leq 35\\ F_{ei} & \left|F_{ei}\right|>35 \end{array},\;F_{ey}\equiv 0,\;M_{ei}=\{\begin{array}{cc} 0 & \left|M_{ei}\right|\leq 20\\ M_{ei} & \left|M_{ei}\right|>20 \end{array},M_{ey}=\{\begin{array}{cc} 0 & \left|M_{ey}\right|\leq 15\\ M_{ey} & \left|M_{ey}\right|>15 \end{array},(i=x,z)$$

where *F*_ei_ and *M*_ei_ represent the force component and the torque component of *F*_e_ along *i*-axis respectively.

The initial docking conditions of two LIDMs are shown in Table 2, where *φ, Φ* and *ψ* represent the yaw angle, pitch angle and roll angle respectively, *X*_e_ and *Z*_e_ represent the transversal offset along ***X***-axis and ***Z***-axis in Cartesian space, while *V*_j_ represents the relative speed of two LIDMs.

### 4.2. Simulation of the Capturing Process of Two LIDMs

**Figure 11.**
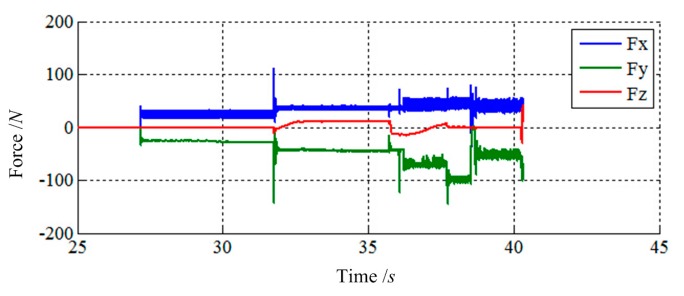
The force tracking results of Case 1.

**Figure 12.**
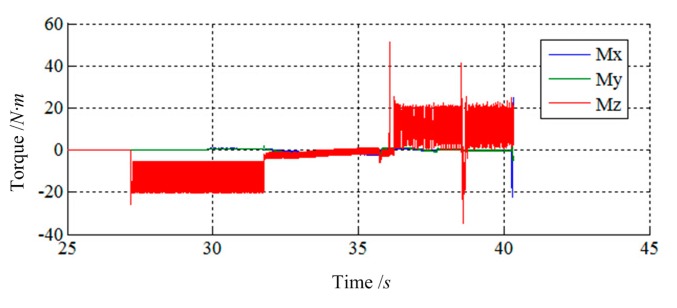
The torque tracking results of Case 1.

**Figure 13.**
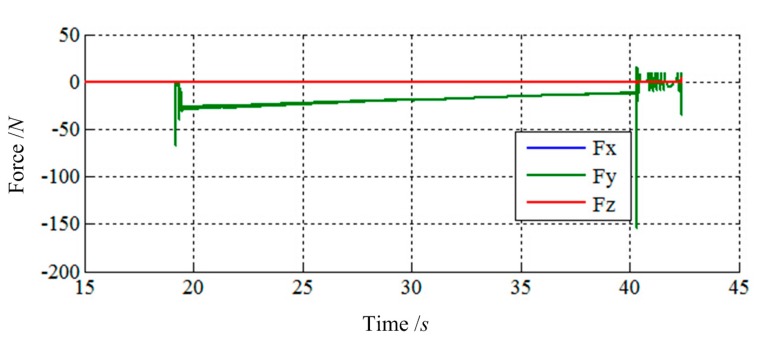
The force tracking results of Case 2.

**Figure 14.**
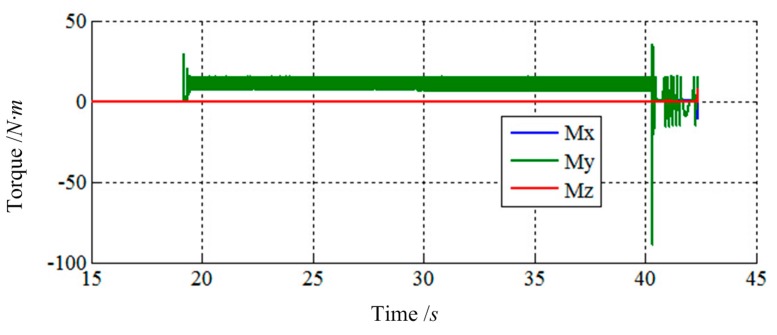
The torque tracking results of Case 2.

**Figure 15.**
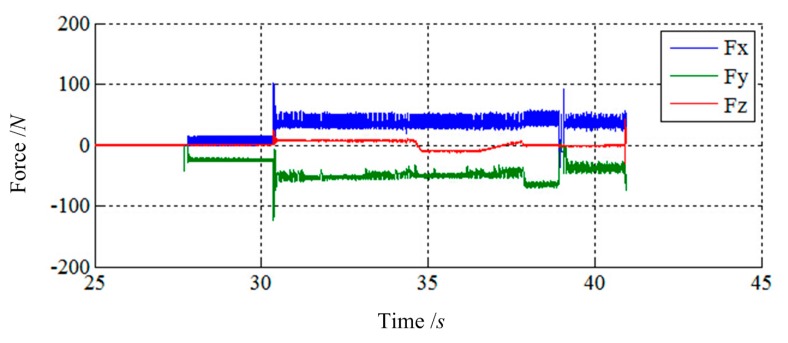
The force tracking results of Case 3.

**Figure 16.**
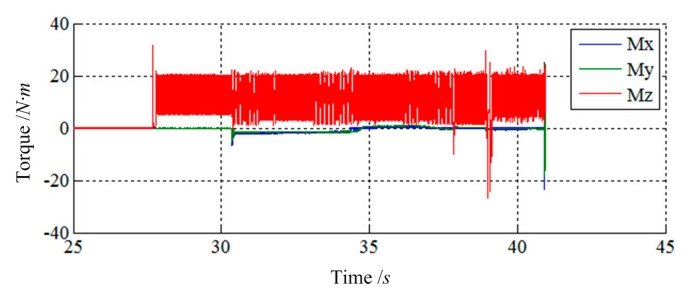
The force tracking results of Case 3.

**Figure 17.**
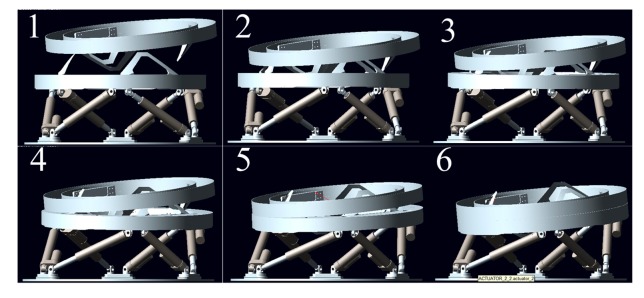
The docking process of Case 4.

**Figure 18.**
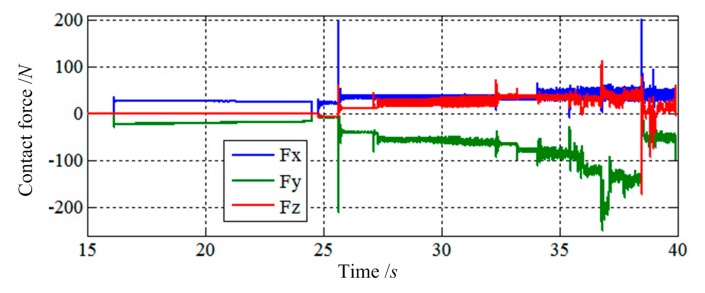
The force tracking results of Case 4.

**Figure 19.**
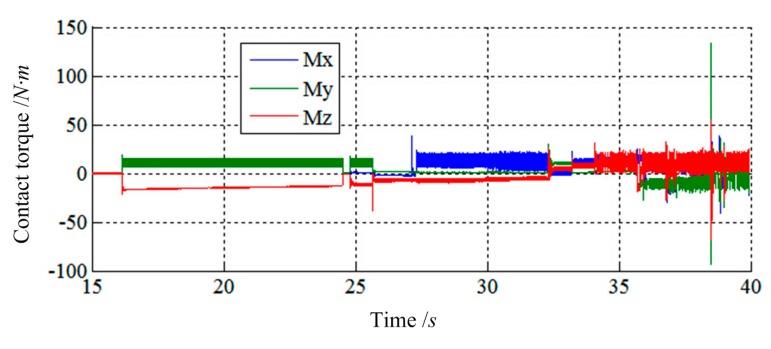
The torque tracking results of Case 4.

The purpose of the simulation is to study whether impedance control based on velocity is suitable for low impact docking of LIDMs or not. In this simulation, the impedance controller controls the velocity of actuators, so the load sensing ring can track the desired Force ***F***_r_ = [0,0,0,0,0,0]^T^ as well as the desired Velocity ***V***_d_ = [0,0,0,0,0,0]^T^. The results are shown in Figures 11, 12, 13, 14, 15, 16, 17, 18 and 19, where *alpha, beta*, and *theta* represent *α, β, θ* respectively.

Figures 11 and 12 show the simulation results of Case 1. The initial contact time of two LIDMs is close to 27 s, and the contact forces do not exceed 150 N and torques do not exceed 60 N·m during the capturing process.

Figures 13 and 14 show the simulation results of Case 2. The initial contact time of two LIDMs is around 19 s, and the contact forces do not exceed 200 N and torques do not exceed 100 N·m during the process of capturing.

Figures 15 and 16 show the simulation results of Case 3. The initial contact time of two LIDMs is close to 27.5 s, and the contact forces do not exceed 200 N and torques do not exceed 40 N·m during the capturing process. Figure 17 shows the docking process of two LIDMs of Case 4. According to Figures 18 and 19, the initial contact time of two LIDMs is close to 16 s, and the contact forces do not exceed 250 N and torques do not exceed 150 N·m during the capturing process. According to Figures 11, 12, 13, 14, 15, 16, 17, 18 and 19, it is easy to find out that there exist some larger forces or torques changing suddenly in all the results of simulations. This phenomenon is triggered by changing the type of contact of two LIDMs from one to another.

According to the simulation results of the four cases, it can be concluded that the contact forces and torques between two LIDMs are instantly translated to adapt to the docking environment with impedance control based on velocity, and the contact forces and torques can be controlled in a small range during the process of capturing. Furthermore, the maximum of contact force and torque are less than 250 N and 150 N·m, respectively, in simulations meeting the requirements, the maximum of contact force and torque are not more than 450 N and 450 N·m, respectively, of docking two LIDMs with low impact forces. Besides, the instantaneous stronger forces are caused by changing the type of contact of two LIDMs from one to another suddenly.

### 4.3. The Influences of Filtering Functions on Control Characteristics

**Figure 20.**
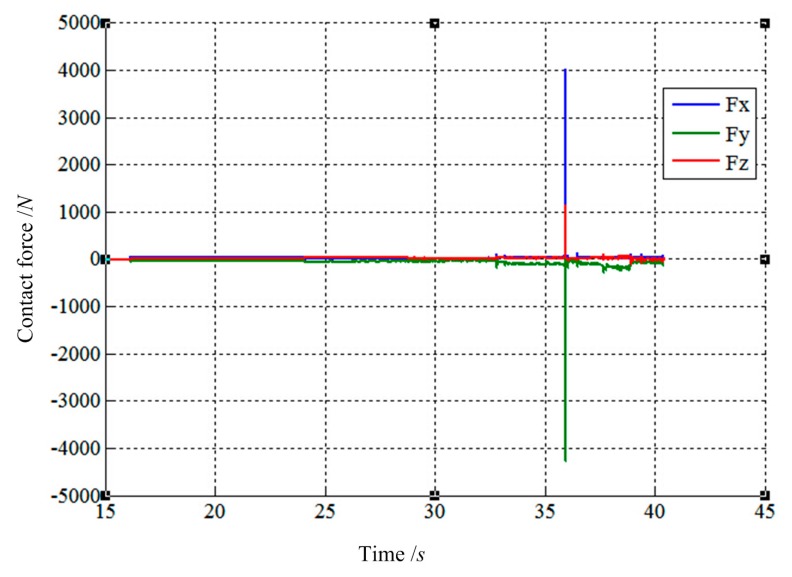
The force tracking results based on Equation (13).

**Figure 21.**
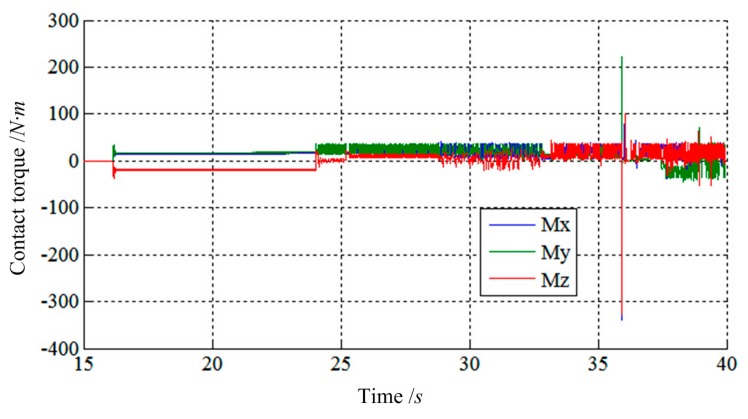
The torque tracking results based on Equation (13).

**Figure 22.**
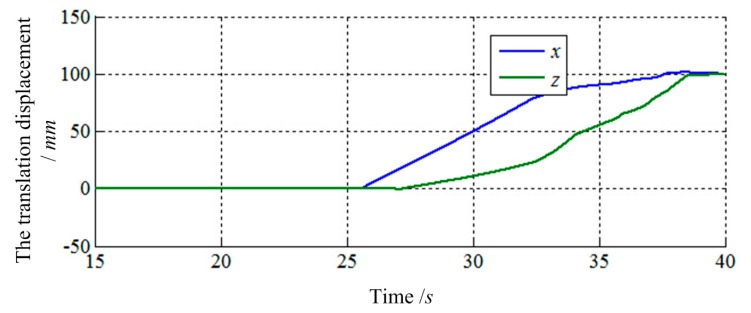
The translation displacement of load sensing ring based on Equation (12).

**Figure 23.**
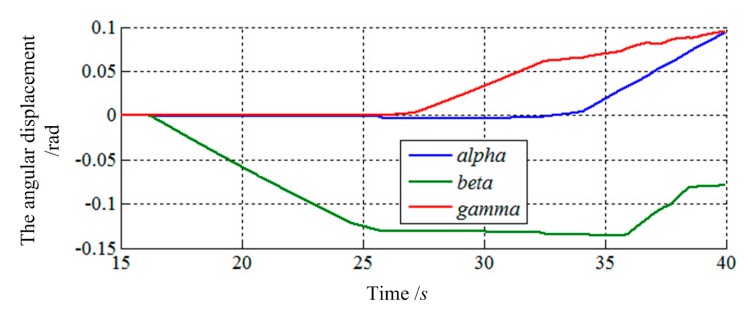
The angular displacement of load sensing ring based on Equation (12).

**Figure 24.**
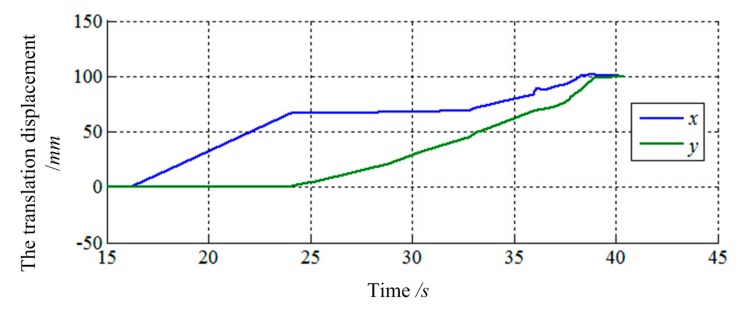
The translation displacement of load sensing ring based on Equation (13).

**Figure 25.**
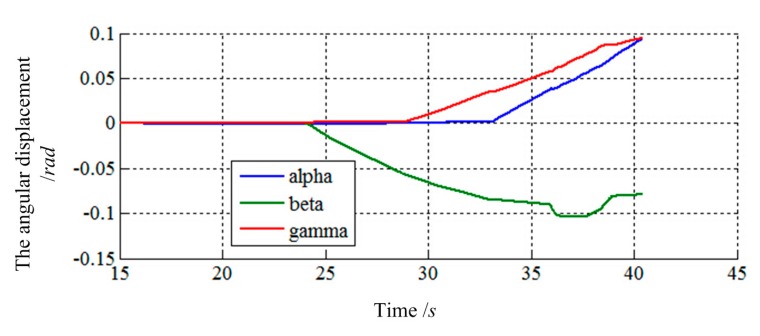
The angular displacement of load sensing ring based on Equation (13).

The purpose of this section is to study how filter affects the control characteristics of LIDM. In this section, another filtering function is given as shown in Equation (13). So the corollary can be obtained by comparing results of two simulations. The results are present in Figures 20, 21, 22, 23, 24 and 25:

(13) $$F_{ei}=\{\begin{array}{cc} 0 & \left|F_{ei}\right|\leq 35\\ F_{ei} & \left|F_{ei}\right|>35 \end{array},\;F_{ey}\equiv 0,\;M_{ej}=\{\begin{array}{cc} 0 & \left|M_{ej}\right|\leq 35\\ M_{ej} & \left|M_{ej}\right|>35 \end{array},\;(i=x,z;j=x,y,z)$$

As shown in Figures 20, 21, 22, 23, 24 and 25, the largest contact force is close to 4000 N while the largest contact torque is more than 300 N·m. Comparing the forces and torques of Figures 20 and 21 with Figures 18 and 19, the largest contact force and torque based on Equation (13) are far more than the values based on Equation (12). Besides, the largest value of force changing suddenly is far more than the value of normal forces during the process of capturing. By comparison, the filter based on Equation (12) is more suitable for the process of capturing, because the sensitivity of forces and torques along three axes of filter has more significant influences during capture.

The final values of translation displacement and angular displacement are shown in Figures 22, 23, 24 and 25. Two simulations are consistent with the same initial docking conditions as shown in Table 2, but the process of moving is entirely different. Without prewired movement path in this method, the motion of load sensing ring is not regular. Thus, how to set the sensitivities about forces and torques of filter along three axes in Cartesian can affect the moving path of the load sensing ring in a manner. In summary, it can be concluded that a filter with suitable filtering functions might greatly improve the capturing process.

## 5. Conclusions

In the capturing simulation by ADAMS and MATLAB, the results of force tracking based on suitable filtering functions are satisfactory. The contact forces and torques of two LIDMs are controlled in a small rang, that the maximum of contact force and torque are not more than 250 N and 150 N·m, respectively, meeting the requirements of docking two LIDMs with low impact forces. In particular, the control system can maintain the contact forces/torques close to expected values when the initial docking conditions are random. Thus, the impedance control algorithm based on velocity is robust and the control system is suitable for LIDM. Although the results are effective, there is still space or room for improvement to reduce the values of contact forces and torques, possibly by choosing more proper impedance parameters or filtering functions.

## Figures and Tables

**Table 1.** The structure parameters of the low impact docking mechanism (LIDM).

***i***	**1**	**2**	**3**	**4**	**5**	**6**
	716.58	−245.25	−471.33	−471.33	−245.25	716.58
***a***_i_/mm	−62.09	−62.09	−62.09	−62.09	−62.09	−62.09
	−130.5	−685.84	−555.32	555.32	685.84	130.5

	722.27	−279.67	−442.6	−442.6	−279.67	722.27
***A***_i_/mm	−102.28	−102.28	−102.28	−102.28	−102.28	−102.28
	−94.09	−672.54	−578.47	578.47	672.54	94.09

	532.25	−532.25	−602.25	−70	70	602.25
***b***_i_/mm	−150	−150	−150	−150	−150	−150
	−388.12	−388.12	−266.88	655	655	−266.88

	70	−70	−662.97	−592.87	592.87	662.87
***B***_i_/mm	0	0	0	0	0	0
	−725	−725	301.88	423.12	423.12	301.88

**Table 2.** The initial docking conditions of two LIDMs.

**Case**	***φ*/Degree**	***Φ*/Degree**	***Ψ*/Degree**	***X*_e_/mm**	***Z*_e_/mm**	***V*_j_/mm/s**
Case 1	0	0	0	100	0	10
Case 2	0	0	20	0	0	10
Case 3	0	10	0	0	0	10
Case 4	5	5	5	100	100	10
